# Direct Crosstalk Between *O*-GlcNAcylation and Phosphorylation of Tau Protein Investigated by NMR Spectroscopy

**DOI:** 10.3389/fendo.2018.00595

**Published:** 2018-10-16

**Authors:** Gwendoline Bourré, François-Xavier Cantrelle, Amina Kamah, Béatrice Chambraud, Isabelle Landrieu, Caroline Smet-Nocca

**Affiliations:** ^1^Univ. Lille, CNRS UMR8576, Unité de Glycobiologie Structurale et Fonctionnelle, Lille, France; ^2^Univ. Paris XI, UMR 1195 Inserm, Le Kremlin Bicêtre, France

**Keywords:** Tau protein, phosphorylation, *O*-GlcNAcylation, crosstalk, NMR spectroscopy

## Abstract

The formation of intraneuronal fibrillar inclusions of tau protein is associated with several neurodegenerative diseases referred to as tauopathies including Alzheimer's disease (AD). A common feature of these pathologies is hyperphosphorylation of tau, the main component of fibrillar assemblies such as Paired Helical Filaments (PHFs). *O*-β-linked N-acetylglucosaminylation (*O*-GlcNAcylation) is another important posttranslational modification involved in regulation of tau pathophysiology. Among the benefits of *O*-GlcNAcylation, modulation of tau phosphorylation levels and inhibition of tau aggregation properties have been described while decreased *O*-GlcNAcylation could be involved in the raise of tau phosphorylation associated with AD. However, the molecular mechanisms at the basis of these observations remain to be defined. In this study, we identify by NMR spectroscopy *O*-GlcNAc sites in the longest isoform of tau and investigate the direct role of *O*-GlcNAcylation on tau phosphorylation and conversely, the role of phosphorylation on tau *O*-GlcNAcylation. We show here by a systematic examination of the quantitative modification patterns by NMR spectroscopy that *O*-GlcNAcylation does not modify phosphorylation of tau by the kinase activity of ERK2 or a rat brain extract while phosphorylation slightly increases tau *O*-GlcNAcylation by OGT. Our data suggest that indirect mechanisms act in the reciprocal regulation of tau phosphorylation and *O*-GlcNAcylation *in vivo* involving regulation of the enzymes responsible of phosphate and *O*-GlcNAc dynamics.

## Introduction

*O*-GlcNAcylation is a dynamic posttranslational modification (PTM) based on addition of a single sugar, the β-D-*N*-acetylglucosamine, on serine and threonine residues of nuclear and cytoplasmic proteins (1). The function of *O*-GlcNAcylation is still not fully understood and may act, at least partially, by regulating phosphorylation. The interplay between both PTMs has been observed upon global elevation of *O*-GlcNAcylation at the phospho-proteome level for which decreases have been detected for some site-specific phosphorylation but also increases, although to a lesser extent, at other sites (2). *O*-GlcNAcylation can compete reciprocally with phosphorylation at the same site or alternatively, both *O*-GlcNAc and phosphate can modify and regulate proximal sites (3). Additionally, the crosstalk between both PTMs may extend to the reciprocal modification of enzymes implicated in phosphorylation and *O*-GlcNAcylation dynamics (2). No consensus sequence has been identified so far for the *O*-GlcNAc modification complicating site mapping. However, frequently occurring motifs exist such as sequences clustering Ser/Thr residues, and bioinformatics tools for *O*-GlcNAc site prediction have been developed that will be of help (4, 5). Recently, a motif frequently occurring in the phosphorylation/*O*-GlcNAcylation crosstalk has been defined involving Ser/Thr proline-directed phosphorylation sites, i.e., sites targeted by proline-directed kinases, that are enriched in the phospho-proteome (6). As a PTM involved in various biological processes, the *O*-GlcNAc modification has been implicated in numerous human diseases including neurodegenerative disorders (7–10).

Variations of *O*-GlcNAc levels in Alzheimer's disease (AD) brain have been detected and are still a matter of debate. It has been shown in some studies that *O*-GlcNAcylation of proteins is lowered in AD brain (9, 11, 12) likely due to decreased glucose uptake/metabolism that is one of the main metabolic changes in aging brain (13). In contrast, overall *O*-GlcNAc levels were also found to be upregulated in AD brain, as associated with a specific increase of *O*-GlcNAc levels of the detergent insoluble protein fraction of cytoskeleton (14) and an imbalance of protein levels involved in the *O*-GlcNAc dynamics as compared to age-matched controls (15). Tau protein, the main component of neurofibrillary tangles (NFTs) (16), one of the two molecular hallmarks of AD with amyloid plaques, is subjected to *O*-GlcNAc modification which was initially estimated to 4 *O*-GlcNAc group per tau molecule on at least 12 Ser/Thr sites (17). It has been shown that tau *O*-GlcNAcylation impairs its phosphorylation and toxicity (11, 18). Furthermore, pharmacological increase of *O*-GlcNAcylation leads to neuroprotective effects and constitutes a potential strategy of treatment of neurodegenerative diseases involving tau pathology.

In AD, tau is hyperphosphorylated (it contains 9–10 moles of phosphate per mol of tau while normal tau contains 2–3 moles of phosphate) (19, 20), undergoes conformational changes (21) and is converted into toxic oligomers and fibrillar aggregates, the straight (SFs) and paired helical filaments (PHFs) (22, 23). Abnormal hyperphosphorylation could be responsible for the conformational changes, oligomerization, fibrillization, and spreading of tau pathology through the brain in specific areas along the course of AD progression (24, 25). Dysregulation of kinase and phosphatase balance is involved in the abnormal phosphorylation of tau but other factors modulate tau phosphorylation. Among them, *O*-GlcNAc has been described in the regulation of tau phosphorylation and reciprocally. *O*-GlcNAc modification is dynamically regulated by only two enzymes, *O*-GlcNAc transferase (OGT) and *O*-GlcNAc hydrolase (OGA), involved in the addition and removal of the GlcNAc moiety, respectively (1, 26–28). Iqbal et al. found that *O*-GlcNAc negatively regulates tau phosphorylation in a site-specific manner in cultured cells, *in vivo* and in metabolically active brain slices (11, 18). In particular, in a mouse model of starvation that mimics impaired glucose metabolism in AD brain, a reduction of tau *O*-GlcNAcylation together with an elevation of tau phosphorylation at specific sites were observed (18). Conversely, inducing hyperphosphorylation of tau with the phosphatase inhibitor okadaic acid leads to a reduction of tau *O*-GlcNAcylation in human neuroblastoma cells together with a reduced transfer into the nucleus (29). Interestingly, hyperphosphorylated tau is less *O*-GlcNAcylated than forms of tau that are less phosphorylated (11, 29). Furthermore, deletion of the *ogt* gene in mice in a neuron-specific manner promotes both an increase of tau amounts and tau hyperphosphorylation, two features that are associated with tau pathology in AD (30). In contrast, stimulating tau *O*-GlcNAcylation by OGT overexpression decreases tau phosphorylation at specific sites (12). Chronic treatment of hemizygous JNPL3 mice carrying the gene for human tau-P301L mutant with Thiamet-G, an OGA inhibitor, leads to a significant reduction of NFTs without altering global tau phosphorylation levels at AD-relevant sites (AT8 and pS422) while *O*-GlcNAc levels were increased significantly, only AT8 immunoreactivity of tangles was reduced (31). The same observation has been made in rTg4510 mice expressing the human tau-P301L protein for which chronic Thiamet-G administration leads to increased tau *O*-GlcNAcylation associated with a decrease of pathological tau aggregates without affecting phosphorylation of non-pathological tau species (32). Hence, OGA inhibition reduces neurofibrillary tangles and neurodegeneration without interfering with tau hyperphosphorylation. Another study has shown that chronic Thiamet-G treatment of tau.P301L transgenic mice although increasing protein *O*-GlcNAcylation has no effect on tau *O*-GlcNAcylation and phosphorylation while improvements of survival and motor deficits have been observed (33). These data suggest that the beneficial effects obtained by elevating *O*-GlcNAc levels may result from enhanced *O*-GlcNAcylation of target proteins other than tau. Furthermore, it was shown that the *O*-GlcNAc modification can directly act on tau aggregation properties by intrinsically decreasing *in vitro* aggregation of recombinant tau induced by heparin without altering its conformation (31, 34).

Recently, however, *O*-GlcNAcylation of endogenous tau has been investigated in mouse models by mass spectrometry identifying a single *O*-GlcNAc modification (at S400) at a low stoichiometry (35) putting into questions the role and mechanism of the *O*-GlcNAc-mediated regulation of tau phosphorylation and pathological aggregation. As *O*-GlcNAcylation could interfere with tau pathology and neurodegeneration, and could be pharmacologically targeted to prevent the pathological formation of toxic tau species (31, 36, 37), a detailed examination of *O*-GlcNAcylation-phosphorylation crosstalk of tau protein is required to fully understand the functional role of tau *O*-GlcNAcylation. NMR proved to be a powerful method to investigate PTMs in intrinsically disordered proteins and disordered regions of globular proteins where posttranslational modifications frequently occur (38–40). Based on this strategy, phosphorylation of tau protein was extensively investigated providing phosphorylation patterns of various kinases in a site-specific manner (41–46). *O*-GlcNAc modification of tau was explored in peptides to identify new O-GlcNAc sites (47) and in a fragment of the C-terminal region containing the S400-*O*-GlcNAc modification to probe potential conformational changes (34). Native chemical ligation afforded a site-specific and quantitative S400-*O*-GlcNAc tau using the expressed protein ligation strategy as a useful tool to decipher the molecular role of *O*-GlcNAcylation in tau functions (48, 49). For the first time, we investigate here the *O*-GlcNAc modification in the entire tau protein obtained by enzymatic activity of OGT and its direct crosstalk with phosphorylation by NMR spectroscopy. We found that tau is not extensively *O*-GlcNAcylated since we detected after *O*-GlcNAc enrichment three major *O*-GlcNAc sites in the C-terminal domain and two *O*-GlcNAc sites of lower level in the proline-rich region. We showed that *O*-GlcNAcylation does not modify phosphorylation of tau by the kinase activity of recombinant ERK2 or rat brain extract while phosphorylation leads to a slight increase of tau *O*-GlcNAcylation by OGT. Our findings contrast with previous models in which phosphorylation and *O*-GlcNAcylation of tau were shown to directly compete *in vivo* in a reciprocal manner, supporting the idea of a more intricate relationship between both PTMs than a direct crosstalk.

## Materials and methods

### Expression and purification of tau protein

BL21 (DE3) *E. coli* strains were transformed with the pET5b plasmid (Novagen, Merck) carrying the longest isoform of *tau* (2N4R isoform) with the S262A mutation for recombinant production. 20 ml of a LB preculture grown at 37°C for 6–8 h were diluted in 2 l of M9 minimal medium. For uniform ^15^N/^13^C-labeling, cells were grown at 37°C in M9 minimal medium containing 2 g/L ^13^C_6_-glucose, 1 g/L ^15^N-ammonium chloride, 0.5 g/L ^15^N/^13^C-Isogro® (Sigma), 1 mM MgSO_4_, MEM vitamin cocktail (Sigma) and 100 mg/L ampicillin. The induction phase was performed by addition of 0.5 mM isopropyl β-D-1-thiogalactopyranoside for 3 h at 37°C. For uniform ^15^N-labeling, the same growth medium was used except that it contained 4 g/L glucose and 0.5 g/L ^15^N-Isogro®. Cells were harvested by centrifugation at 5,000 × g for 30 min and the pellet was resuspended in 50 mM NaH_2_PO_4_/Na_2_HPO_4_, pH 6.2, 2.5 mM EDTA, 2 mM DTT and 0.5% Triton X-100 supplemented with a Complete^TM^ protease-inhibitor cocktail. The lysate was obtained by homogenizing this suspension using a high-pressure homogenizer followed by centrifugation at 30,000 × g for 30 min at 4°C. The extract was incubated at 75°C for 15 min and centrifuged at 4,000 × g for 20 min at 4°C as a first purification step. The tau protein was then purified by cation-exchange chromatography (HiTrap SP HP 5 ml, GE Healthcare) in 50 mM NaH_2_PO_4_/Na_2_HPO_4_, pH 6.4, 2 mM EDTA (buffer A). After loading the extract onto the column and column washing with buffer A, the protein was eluted with a gradient from 0 to 50% buffer B (buffer A supplemented with 2M NaCl) over 20 ml (i.e. 4 column volumes). Fractions were analyzed by SDS-PAGE and fractions containing tau protein with highest purity were pooled together for a next step of purification by RP-HPLC on a Zorbax 300SB-C8 semi-preparative 9.4 × 250 mm column (Agilent) equilibrated in a solution of 0.1% TFA:2% acetonitrile. Proteins were eluted by a 30-min linear gradient from 16% to 40% acetonitrile at 5 ml/min. Fractions containing full-length tau protein are pooled, lyophilized, and buffer-exchanged in 50 mM ammonium bicarbonate (HiPrep 26/10 desalting, GE Healthcare) to avoid acidification of solutions due to residual TFA, and lyophilized again before storage at −20°C until further use (50). Using this procedure, the yield of tau protein was 10 mg per liter of culture.

### Expression and purification of the nucleocytoplasmic form of OGT

BL21 (DE3) *E. coli* strains were transformed with the pET24d plasmid (Novagen, Merck) carrying the nucleocytoplasmic isoform of human *ogt* for the recombinant production of ncOGT (51). Freshly plated bacteria were picked up to inoculate a 20 ml culture which was grown at 37°C overnight. The 20 ml culture then inoculated a 2 l culture in LB rich medium that was grown at 37°C for 3 h until O.D. at 600 nm reached 0.6, then the culture was cooled down to 16°C. The protein induction was performed at 16°C for 24 h upon addition of 0.2 mM IPTG. Cells were harvested at 6,000 × g for 30 min at 4°C and the pellet was resuspended in 100 ml extraction buffer (50 mM KH_2_PO_4_/K_2_HPO_4_ pH 7.60, 250 mM NaCl, 40 mM imidazole, 5% glycerol, 1% Triton X-100 complemented with a 0.3 mg DNase I and EDTA-free protease inhibitor cocktail). Cell lysis was performed with a high-pressure homogenizer at 4°C followed by a brief sonication step. Soluble proteins were isolated from the bacterial extract by centrifugation at 30,000 × g for 30 min at 4°C. An affinity purification step was performed on a 1 ml-HiTrap Chelating column (GE Healthcare) loaded with Ni^2+^ ions. The full-length OGT was eluted with 250 mM imidazole after a truncated form was eluted with 110 mM imidazole. Homogenous fractions containing the full-length recombinant ncOGT as checked on a 10% polyacrylamide-SDS gel were pooled (in a final volume 10 ml) and dialysed at 4°C overnight in 2 l of sample buffer (50 mM KH_2_PO_4_/K_2_HPO_4_ pH 7.60, 150 mM NaCl, 1 mM EDTA). After dialysis, the sample was supplemented with 0.5 mM THP and stored at −80°C. Starting from a 2 l-culture, we obtained 5 mg of ncOGT. The *O*-GlcNAc-transferase activity of the recombinant ncOGT was checked on a peptide substrate from casein kinase II (CKII) indicating that the recombinant ncOGT is fully active as it provides an *O*-GlcNAcylated CKII peptide with 95% *O*-GlcNAcylation after 30 min incubation at 37°C (52, 53).

### *In vitro O*-GlcNAc transferase reaction of tau by recombinant ncOGT

10 mg of ^15^N/^13^C-labeled tau protein (M.W. 48,070 kDa) were solubilized at 0.83 mM in a solution of ncOGT at 10 μM in a final volume of 250 μl of reaction buffer (50 mM KH_2_PO_4_/K_2_HPO_4_ pH 7.6, 150 mM NaCl, 1 mM EDTA, 0.5 mM THP, 12.5 mM MgCl_2_, 10 mM UDP-GlcNAc). As a negative control, a reaction was carried out in the same conditions without UDP-GlcNAc. The *O*-GlcNAcylation reactions were performed at 31°C for 3 days. *O*-GlcNAcylation of tau was checked after incubation periods of 6 and 24 h but higher *O*-GlcNAcylation levels were obtained for longer timescale. After 3 days, the reaction mixtures were heated at 75°C, centrifuged and purified by RP-HPLC on a Zorbax 300SB-C8 semi-preparative column (9.4 × 250 mm, Agilent) equilibrated in buffer A (0.1% TFA: 2% acetonitrile). Proteins were eluted with a linear gradient from 25 to 50% of buffer B (0.1% TFA: 80% acetonitrile) of 25 min. Fractions enriched in *O*-GlcNAc were identified by MALDI-TOF MS and Click-iT^TM^ *O*-GlcNAc labeling and TAMRA detection kits (Molecular Probes) on SDS-PAGE following the manufacturer's instructions (Figure S1). Fractions containing protein with low levels of *O*-GlcNAcylation were pooled, lyophilized, desalted in 50 mM ammonium bicarbonate (HiTrap Desalting 5 ml, GE Healthcare) prior to another round of *O*-GlcNAcylation and purification. At the end, the *O*-GlcNAc-enriched fractions corresponding to the two rounds of *O*-GlcNAcylation/purification were lyophilized. This procedure affords about 3-3.5 mg of *O*-GlcNAc enriched tau (hereafter referred to as tau-*O*-GlcNAc) from 10 mg of tau. For an estimation of site-specific *O*-GlcNAc modification by NMR spectroscopy, this procedure was performed twice with two distinct batches of ncOGT.

^15^N-tau phosphorylated by ERK2, ^15^N/^13^C-tau phosphorylated by a rat brain extract or ^15^N-tau as a control were subjected to *O*-GlcNAcylation reactions at 0.4 mM in a solution of ncOGT at 10 μM in OGT reaction buffer (50 mM KH_2_PO_4_/K_2_HPO_4_ pH 7.6, 150 mM NaCl, 1 mM EDTA, 0.5 mM THP, 12.5 mM MgCl_2_, 10 mM UDP-GlcNAc) at 31°C for 2 days. As a negative control, reactions were carried out in the same conditions without UDP-GlcNAc. To stop the *O*-GlcNAc reactions, the reaction mixtures were heated at 75°C and centrifuged at 16,000 × g for 15 min at 4°C. Then, the proteins were purified by reverse phase chromatography on a Zorbax 300SB-C8 semi-preparative column (9.4 × 250 mm, Agilent) for NMR analyses. Unlike non-phosphorylated tau, no *O*-GlcNAc enrichment was observed along the elution step of reverse phase chromatography for phosphorylated tau proteins as detected by MALDI-TOF MS. *O*-GlcNAcylation levels of phosphorylated vs. non-phosphorylated tau were quantitatively assessed using the Click-iT^TM^ *O*-GlcNAc labeling and TAMRA detection kits following the manufacturer's instructions. 20 and 5 μg of proteins dissolved at ≈ 2 μg/μl in Laemmli sample buffer 3X were loaded on SDS-PAGE for TAMRA detection and Coomassie staining, respectively. Quantification of TAMRA fluorescence was performed with ImageQuant^TM^ LAS 4000 (GE Healthcare).

For the preparation of NMR samples as well as for the phosphorylation assays, the amount of various protein samples in their *O*-GlcNAcylated and non *O*-GlcNAcylated forms was calculated based on the peak integration at 280 nm assuming that the molar extinction coefficient is the same between *O*-GlcNAc and non *O*-GlcNAc proteins.

### MALDI-TOF MS analyses of tau proteins

MALDI-TOF mass spectrometry analyses were performed with sinapinic acid matrix on an Axima Assurance mass spectrometer (Shimadzu) in a linear positive ion mode.

### Expression and purification of ERK2 and MEK3 kinases and phosphorylation of tau

Recombinant mitogen-activated protein kinase ERK2 and a constitutively active mutant of mitogen-activated protein kinase kinase MEK3 (hereafter referred to as MEK3), that phosphorylates and activates ERK2, were produced as described previously (41, 46, 50). Briefly, ERK2 carrying a polyhistidine tag and MEK3 carrying a GST tag were purified by affinity chromatography from 1 l culture in LB after an induction phase at 30°C for 4 h with 0.5 mM IPTG. ERK2 was purified on a 1-ml HisTrap HP column (GE Healthcare) equilibrated in 50 mM NaH_2_PO_4_/Na_2_HPO_4_, pH 7.5, 300 mM NaCl, 20 mM imidazole (buffer A) and eluted with a buffer containing 50 mM NaH_2_PO_4_/Na_2_HPO_4_, pH 7.5, 300 mM NaCl, 250 mM imidazole (buffer B). MEK3 was purified on glutathione sepharose beads (2 ml) equilibrated in 50 mM Tris.Cl pH 7.5, 300 mM NaCl, 1 mM EDTA (buffer C). After loading of the bacterial extract at 4°C for 3 h and washing beads 5 times with 10 ml of buffer C then 3 times with 10 ml of phosphorylation buffer (50 mM Hepes. KOH, pH 7.8, 12.5 mM MgCl_2_, 50 mM NaCl, 1 mM EGTA), GST-MEK3 was left on resin beads for phosphorylation reactions.

For the phosphorylation reactions, fractions containing ERK2 eluted from the affinity chromatography step was buffer-exchanged in phosphorylation buffer on a 5 ml Zeba^TM^ Spin desalting column (Thermo Fischer) and mixed with GST-MEK3 beads.

ERK2 activation and tau phosphorylation were performed simultaneously. ^15^N/^13^C-tau-*O*-GlcNAc (3 mg) that was previously *O*-GlcNAcylated by OGT and ^15^N-tau (10 mg) as a control were dissolved at 100 μM with ERK2 and MEK3 in phosphorylation buffer complemented with 6.25 mM ATP and 1 mM DTT and incubated at 37°C for 5 h. Phosphorylation reactions were stopped by centrifugation of the reaction mixtures at 4,000 × g for 10 min to precipitate the resin beads and incubating the supernatant at 75°C for 15 min followed by centrifugation at 4,000 × g for 15 min. Then, the supernatants were buffer-exchanged in 50 mM ammonium bicarbonate on a 5 ml HiTrap Desalting column (GE Healthcare) and lyophilized. Prior to NMR analyses, phosphorylation was qualitatively checked by a shift in the apparent mobility of the protein band on SDS-PAGE and by MALDI-TOF MS (41, 46). Phosphorylation of tau with activated ERK2 was performed twice with two different batches of ERK2 and MEK3. An average phosphorylation level of 17 ± 2 phosphate per tau molecule was determined by mass spectrometry on intact proteins.

### Phosphorylation of tau by a rat brain extract

A rat brain extract was prepared from adult Sprague-Dawley rats by homogenizing whole brains (~2 g) in 5 mL of homogenizing buffer, containing 10 mM Tris-HCl, pH 7.4, 5 mM EGTA, 2 mM DTT, 1 μM okadaic acid (OA), 20 μg/mL Leupeptin, and 40 mM Pefabloc®. Insoluble material was precipitated by ultracentrifugation at 100,000 × g and 4°C for 1 h. The supernatant was directly used for its kinase activity. Total protein concentration was estimated at 9 mg/mL by a BCA colorimetric assay. ^15^N/^13^C-tau and ^15^N/^13^C-tau-*O*-GlcNAc were dissolved at 10 μM in 4.5 mL of phosphorylation buffer containing 40 mM Hepes.KOH, pH 7.3, 2 mM MgCl_2_, 5 mM EGTA, 2 mM DTT, 2 mM ATP, 1 μM OA and supplemented with a Complete™ protease-inhibitor cocktail. PUGNAc (Sigma), an inhibitor of β-hexosaminidases and *O*-GlcNAc hydrolase, was added at 10 μM in the phosphorylation reaction of tau-*O*-GlcNAc to prevent deglycosylation. The proteins were incubated with 500 μL of rat brain extract at 37°C for 24 h. Then, reactions were stopped by heating the mixture at 75°C for 15 min followed by centrifugation at 16,000 × g for 20 min. The supernatants were buffer-exchanged in 50 mM ammonium bicarbonate and the proteins were lyophilized. Evaluation of protein phosphorylation was performed by SDS-PAGE and MALDI-TOF MS, and then the phosphorylation sites were determined by NMR analyses.

### NMR spectroscopy

NMR experiments were performed at 293K on Bruker 900 MHz Avance NEO and 600 MHz Avance III HD spectrometers (Bruker, Karlsruhe, Germany) equipped with a 5-mm TCI and QCI cryogenic probes, respectively. For NMR experiments, tau protein samples were dissolved at a concentration of 200–400 μM in a buffer containing 50 mM NaH_2_PO_4_/Na_2_HPO_4_ pH 6.5, 25 mM NaCl, 2.5 mM EDTA, 1 mM DTT, 10% D_2_O either in a volume of 200 μl (in 3 mm tubes) or in 300 μl (in Shigemi tubes). ^1^H spectra were calibrated with 0.25 mM of sodium 3-(trimethylsilyl) propionate-2,2',3,3'-d_4_ as internal standard. ^1^H spectra were acquired with 64 scans and 32 dummy scans, and a spectral windows of 14 ppm centered on 4.7 ppm sampled with 32k points. For 2D and 3D experiments, a spectral window of 14 ppm centered on 4.7 ppm was used for the proton dimension.

^1^H-^15^N HSQC spectra were recorded with 64 scans per increment and 16 dummy scans with 3072 and 512 points in the proton and nitrogen dimensions, respectively, and with a window of 25 ppm centered on 118 ppm for the nitrogen dimension. Heteronuclear experiments were recorded with a WATERGATE sequence for water suppression and a double INEPT (INsensitive nuclei Enhanced by Polarization Transfer) for sensitivity improvement. All experiments were acquired with a recycle delay of 1s.

Assignment of modified (phosphorylated or *O*-GlcNAcylated) tau proteins required the acquisition of three-dimensional NMR experiments on ^15^N/^13^C-labeled tau samples at 293K. The HNCACB and HN(CO)CACB experiments were recorded with 32 scans per increment and 16 dummy scans, with 2048, 90, and 120 points in the proton, nitrogen and carbon dimensions, respectively, and with a window of 25 and 60 ppm centered on 118 ppm and 44 ppm for the nitrogen and carbon dimensions, respectively. The HN(CA)NNH experiment was recorded with 24 scans per increment and 16 dummy scans, with 2048, 120, and 180 points in the proton and both nitrogen dimensions, respectively, and with a window of 25 ppm centered on 118 ppm for the nitrogen dimensions.

*O*-GlcNAc and phosphorylation levels were determined for individual Ser/Thr residues based on intensity of their respective N-H correlations of their modified and non-modified forms from the ^1^H-^15^N HSQC experiment.

## Results

### Identification of tau *O*-GlcNAc sites by NMR spectroscopy

We first addressed the identification of *O*-GlcNAc sites in full-length tau provided by the enzymatic activity of ncOGT *in vitro* using NMR spectroscopy. The enzymatic *O*-GlcNAcylation of uniformly ^15^N/^13^C-labeled tau protein was performed as described for peptide substrates previously (47, 54). After *O*-GlcNAc enrichment of tau protein by reverse phase chromatography, the MALDI-TOF MS analysis of the tau-*O*-GlcNAc sample indicated an addition of 1.9 GlcNAc per tau molecule taking into account a m/z increment of +203 per GlcNAc.

**Figure 1:**
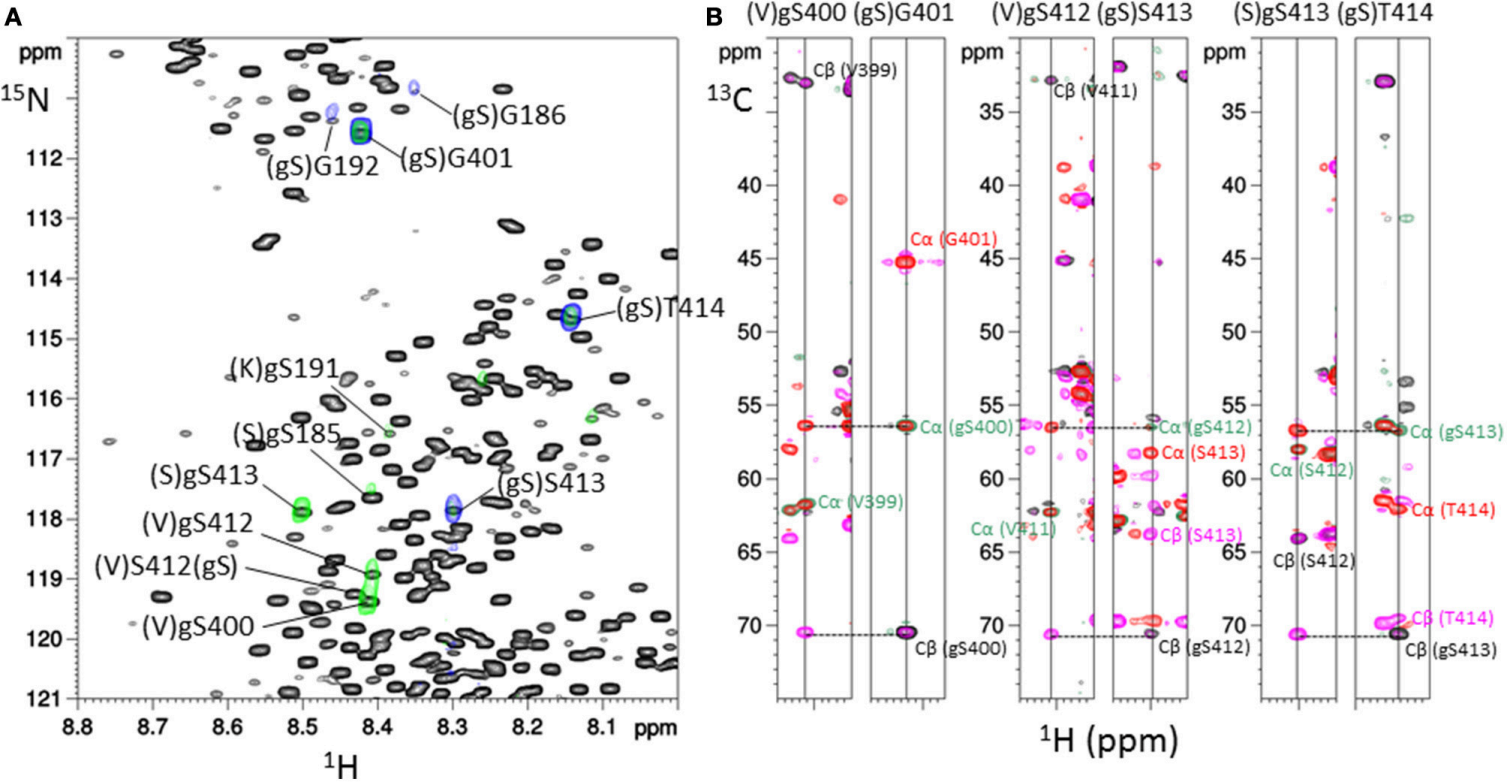
Assignment strategy of *O*-GlcNAc sites in full-length tau protein. **(A)** Superimposition of ^1^H-^15^N HSQC spectrum of tau-*O*-GlcNAc (black) and ^1^H-^15^N planes extracted from HNCACB (green) and HNcoCACB (blue) three-dimensional experiments at a ^13^C value of 70.6 ppm corresponding to the Cβ resonance of *O*-GlcNAc serine, highlighting the resonances of the *O*-GlcNAc sites (i residues) and their (i+1) residues, respectively. **(B)** ^1^H-^13^C planes from HNCACB (red and pink for the Cα and Cβ resonances, respectively) and HNcoCACB (green and black for the Cα and Cβ resonances, respectively) extracted at ^15^N values corresponding to resonances of residues indicated above and shown in the ^1^H-^15^N HSQC experiment in **(A)**.

In the ^1^H-^15^N HSQC experiment, additional signals were observed as compared to non-modified tau spectrum as a control indicating either *O*-GlcNAc sites or residues located in their neighborhood (Figure S2). The NMR-based detection of *O*-GlcNAc sites within tau protein further made use of the chemical shift changes of ^13^Cα and ^13^Cβ values upon Ser/Thr *O*-GlcNAcylation (38, 47, 54). Acquisition of three-dimensional HNCACB and HNcoCACB experiments afforded ^13^C chemical shifts of each residue (i) and its preceding residue (i-1) in the protein sequence allowing amino acid identification in the context of an intrinsically disordered protein such as tau. By selecting ^13^Cα and ^13^Cβ values of 56.5 ppm and 70.6 ppm, respectively, ^1^H-^15^N planes were extracted from HNCACB corresponding to ^1^H-^15^N HSQC sub-spectra of *O*-GlcNAc Ser residues (Figure 1) (54). The same processing of HNcoCACB afforded (i+1) residues of *O*-GlcNAc serines (Figure 1). The HNcaNNH experiment that provides ^15^N values of (i-1) and (i+1) residues confirmed the identification of *O*-GlcNAc sites.

**Figure 2:**
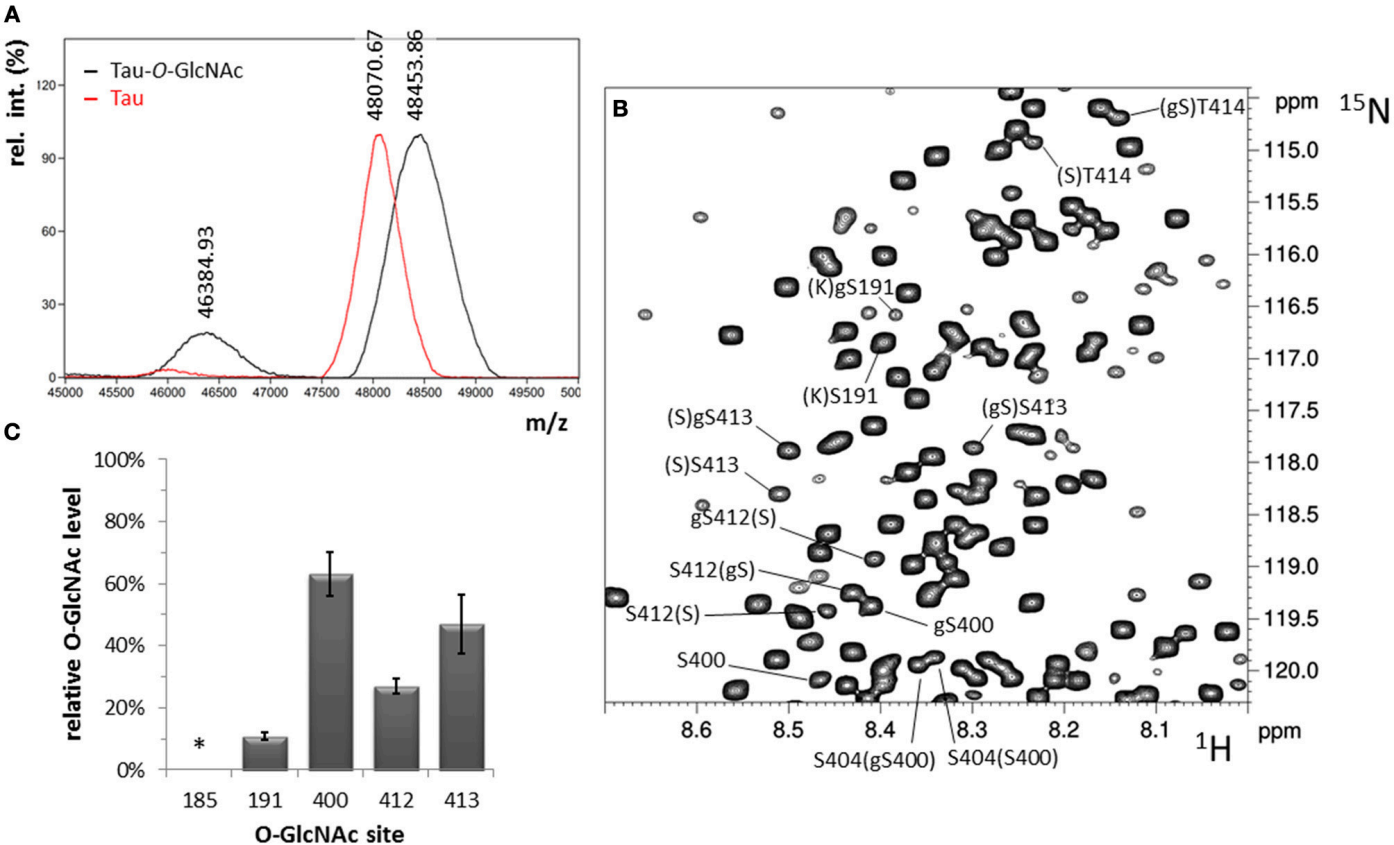
*O*-GlcNAcylation of tau. **(A)** Overall *O*-GlcNAcylation level determined by MALDI-TOF MS. **(B)** ^1^H-^15^N HSQC spectrum of tau-*O*-GlcNAc with assignment of *O*-GlcNAc sites. **(C)** Site-specific *O*-GlcNAc levels determined by NMR spectroscopy. The *O*-GlcNAc level indicated by an asterisk cannot be calculated due to signal overlap.

With this strategy, three *O*-GlcNAc Ser residues were identified in the C-terminal region of tau at position S400, S412, and S413 (Table S1). No threonine residues were detected with the same strategy taking into account ^13^C chemical shifts changes of Thr residues upon *O*-GlcNAcylation. Resonance intensities in the two-dimension ^1^H-^15^N HSQC experiment corresponding to *O*-GlcNAc and non-modified forms gave the *O*-GlcNAcylation level for each *O*-GlcNAc site providing a quantitative *O*-GlcNAc pattern of tau protein with a relative distribution of the mono-*O*-GlcNAc species of 63 ± 7% for S400, 27 ± 2% for S412 and 47 ± 9% for S413 (Figure 2). Given the relative abundance of both gS412 and gS413 at vicinal positions, a di-*O*-GlcNAc specie would have a theoretical relative abundance of 13% and should be detected. However, we found neither (gS)gS413 nor another (V)gS412 resonance indicating the presence of a di-*O*-GlcNAc form. This suggests that both *O*-GlcNAc modifications are mutually exclusive. Additionally, two (gS)G motifs were detected in the ^1^H-^15^N plane extracted from the HNcoCACB experiment at the ^13^Cβ chemical shift of Ser-*O*-GlcNAc pointing to two *O*-GlcNAc sites, S185 and S191, in the proline-rich domain that were also detected in the HNCACB experiment (Table S1). S191 *O*-GlcNAc level was estimated to 11 ± 1% while the level of S185 *O*-GlcNAc could not be determined based on signals of S185 due to signal overlap of both the *O*-GlcNAc and non-modified forms, but seems to be of the same order of magnitude than S191-*O*-GlcNAc based on the resonances of G186 in the *O*-GlcNAc and non-glycosylated forms. Other resonances were detected in the HSQC indicating the presence of additional *O*-GlcNAc sites but they were not unambiguously assigned due to their low intensity (Figure 1) suggesting that they were *O*-GlcNAcylated at a level inferior to 5%.

### Phosphorylation improves direct tau *O*-GlcNAcylation by OGT

**Figure 3:**
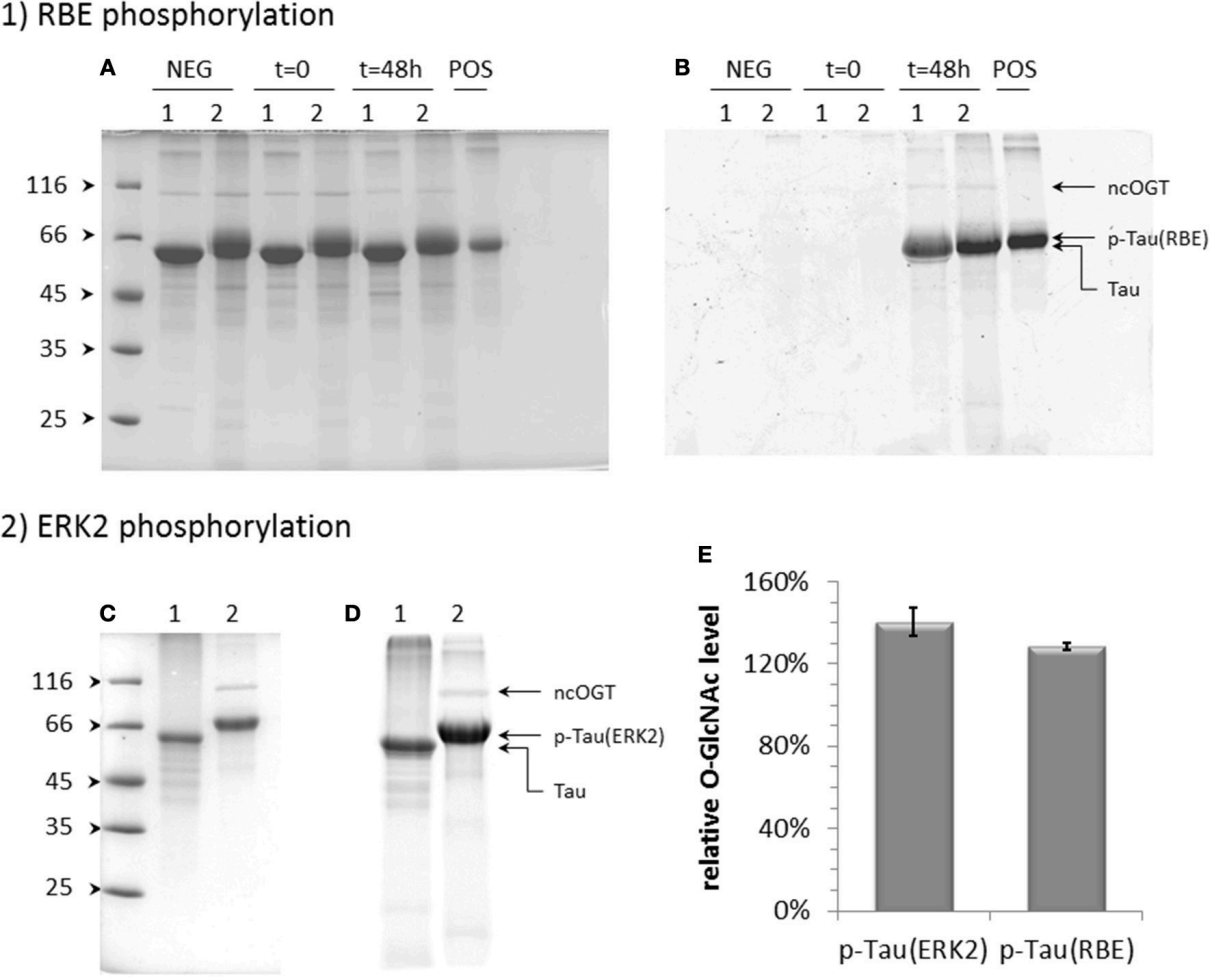
Effect of phosphorylation on tau *O*-GlcNAcylation by OGT. Effect of phosphorylation by kinase activity of a rat brain extract (RBE) **(A,B)** or activated ERK2 **(C,D)** on tau *O*-GlcNAcylation detected by TAMRA fluorescence in polyacrylamide gel **(B,D)** after *O*-GlcNAc labeling. Protein loading on SDS-PAGE was checked by Coomassie staining **(A,C)**. Lanes 1 correspond to unmodified tau and lanes 2 to phosphorylated tau incubated for 48 h at 31°C with OGT and 10 mM UDP-GlcNAc; *t* = 0 is given for starting reaction of RBE phosphorylated tau as a control of protein degradation during the *O*-GlcNAc transferase reaction; NEG indicates *O*-GlcNAc transferase reaction performed in absence of UDP-GlcNAc as a negative control; POS corresponds to tau-*O*-GlcNAc sample as a positive control of *O*-GlcNAc labeling and TAMRA-alkyne click reactions. **(E)** Quantification of *O*-GlcNAc relative levels of tau proteins phosphorylated either by ERK2, p-Tau(ERK2), or by RBE, p-Tau(RBE), normalized on the signal of non-phosphorylated tau.

**Figure 4:**
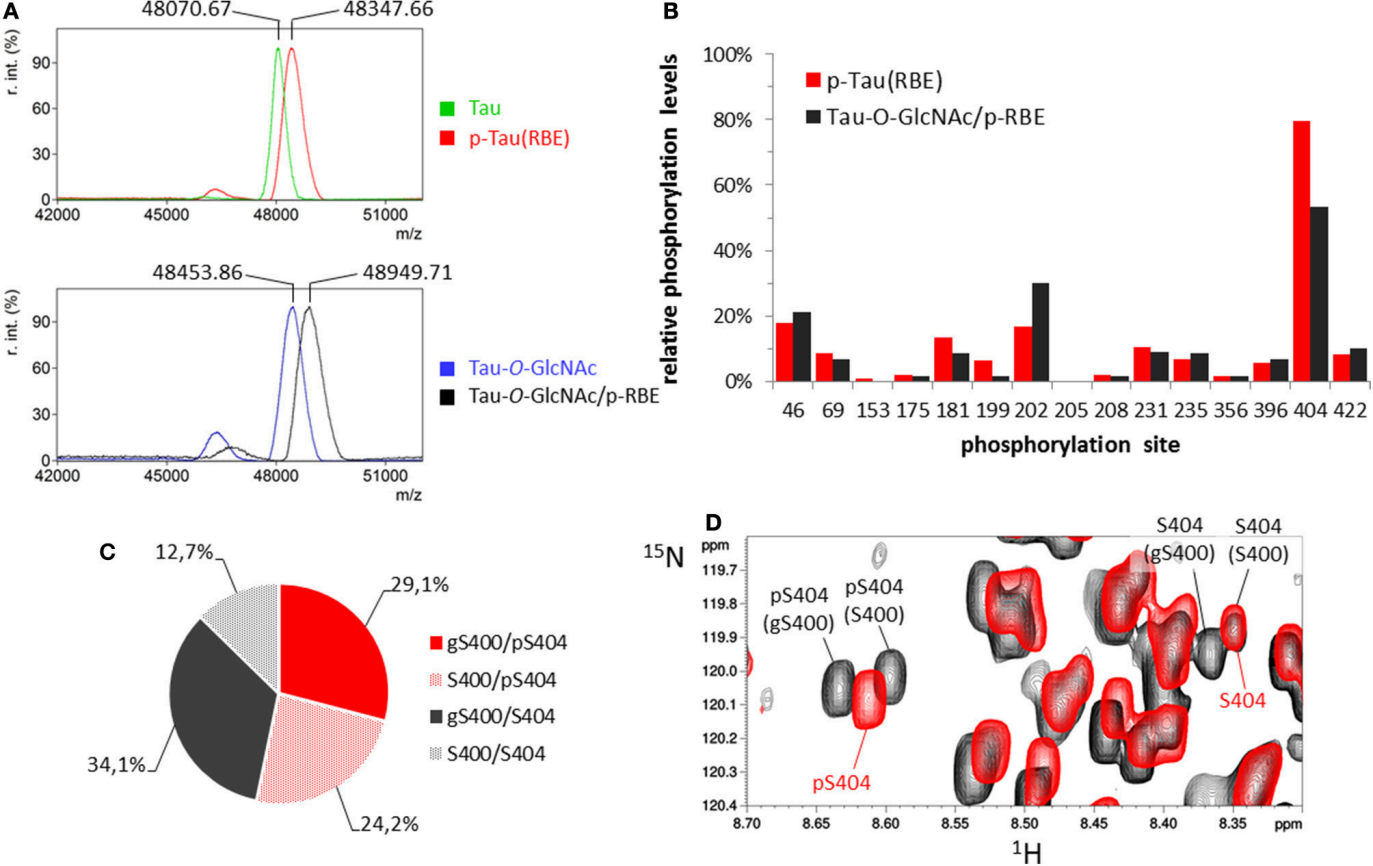
*O*-GlcNAc-mediated regulation of tau phosphorylation. **(A)** MALDI-TOF mass spectra of ^15^N/^13^C-tau before (green) and after phosphorylation by RBE (red), and ^15^N/^13^C-tau-*O*-GlcNAc before (blue) and after phosphorylation by RBE (black). **(B)** Phosphorylation pattern of tau (red) and tau-*O*-GlcNAc (black) phosphorylated by RBE. **(C,D)** Distribution of phosphorylated and *O*-GlcNAcylated species at the C-terminal S400/S404 sites. **(C)** Phosphorylated S404 (pS404) is depicted in the pie chart by red sectors and non-phosphorylated S404 by gray sectors; *O*-GlcNAcylated S400 (gS400) is depicted by plain sectors and non-*O*-GlcNAcylated S400 by dotted sectors. *O*-GlcNAcylation of S400 was estimated to 63% and S404 phosphorylation in tau-*O*-GlcNAc/p-RBE to 53%. **(D)** Superimposition of ^1^H-^15^N HSQC spectra of p-tau(RBE) (red) and tau-*O*-GlcNAc/p-RBE (black) showing the resonances of pS404 and S404.

As both C-terminal and proline-rich domains where *O*-GlcNAc sites were found are enriched in proline-directed Ser/Thr phosphorylation sites, *O*-GlcNAcylation of phosphorylated tau was also evaluated in a similar manner as the non-modified form although *O*-GlcNAc-enrichment of the phosphorylated tau failed, probably due to PTM heterogeneity of the sample. We first address the effect of a phosphorylation pattern obtained by kinase activity of a rat brain extract complemented by ATP in which phosphatase activity was blocked by okadaic acid (41, 55, 56). The kinase activity was moderate (≈4.5 ± 1 mol of phosphate per mol of tau protein) as determined by mass spectrometry (see Figure 4A) as compared to patterns previously described. Phosphorylated tau was *O*-GlcNAcylated by OGT as well as non-phosphorylated tau as a control and overall *O*-GlcNAc levels were determined by mass spectrometry (Figure S3) and selective enzymatic labeling of tau-*O*-GlcNAc by azide-modified GalNAc and copper-mediated click chemistry with an alkyne derivative of TAMRA for detection of modified tau protein. The *O*-GlcNAc transferase reaction of phosphorylated tau with recombinant OGT resulted in a slightly higher level of *O*-GlcNAcylation than unmodified tau (Figures 3A,B,E).

We next investigated the effect of a higher level of phosphorylation on the *O*-GlcNAc transferase activity of OGT. Hyperphosphorylation was obtained with kinase activity of recombinant ERK2 with an overall level of 19 ± 1 phosphate per tau molecule (Figure S4A). The phosphorylation pattern determined by NMR spectroscopy was similar to those previously described including all proline-directed Ser/Thr in tau sequence (41, 55). A quantitative phosphorylation was obtained for most sites (Figure S4B). In particular, S396, S404, and S422 in the C-terminal domain were phosphorylated at high levels of 94, 97, and 85%, respectively, and S191 in the proline-rich domain at 68%. In this hyperphosphorylated tau sample, the *O*-GlcNAc transferase activity of OGT was 1.4-fold higher than in unphosphorylated tau (Figures 3C-E) which is a higher activity than those measured with the phosphorylation pattern of the rat brain extract for which phosphorylation level was lower. At the residue level, only a splitting of pS404 resonance was observed among the resonances of phospho-residues in the ^1^H-^15^N HSQC spectrum upon *O*-GlcNAcylation on top of tau phosphorylation by ERK2 indicating that pS404 amide resonance is perturbed by proximal *O*-GlcNAcylation, likely S400 *O*-GlcNAcylation. This effect on pS404 in contrast to pS396 resonance revealed a specific interaction with S400 *O*-GlcNAcylation which could be due to local conformational changes induced either by *O*-GlcNAcylation, phosphorylation or both of them. Except resonances corresponding to *O*-GlcNAc sites previously identified in tau, no additional resonance was detected in the spectrum pointing to new *O*-GlcNAc sites. Together our data indicate that phosphorylation leads to an overall moderate improvement of OGT activity on tau protein without additional *O*-GlcNAc sites that can be detected by NMR.

### Regulation of tau phosphorylation by *O*-GlcNAcylation

We first determined the effect of *O*-GlcNAcylation on kinase activity of a rat brain extract complemented by okadaic acid to inhibit phosphatase activities (41, 55, 56). Additionally, PUGNAc, a β-hexosaminidase and *O*-GlcNAcase inhibitor (57), was added to prevent *O*-GlcNAc hydrolysis during incubation of tau-*O*-GlcNAc protein with the extract. The kinase activity barely increased in tau-*O*-GlcNAc compared to unmodified tau as detected by mass spectrometry with ≈6 ± 0.5 mol of phosphate per mol of tau protein which is slightly higher than the overall level of phosphorylation obtained with non-modified tau (Figure 4A). Furthermore, phosphorylation patterns of tau and tau-*O*-GlcNAc were examined using high resolution NMR spectroscopy to evaluate direct modulation of site-specific phosphorylation by *O*-GlcNAcylation (Figure 4B and Figure S5). We found no major change in the phosphorylation pattern of tau except for S404 and S202. S404 phosphorylation was slightly decreased in tau-*O*-GlcNAc likely due to its proximity with the S400-*O*-GlcNAc site of highest occupancy. As described previously, the resonances of pS404 and S404 were splitted in tau-*O*-GlcNAc/p-RBE ^1^H-^15^N HSQC spectrum due to incomplete S400 *O*-GlcNAcylation which induces a modification of the chemical environment of S404 residue in both its phosphorylated and non-phosphorylated forms. S404 phosphorylation of 53% in tau-*O*-GlcNAc/p-RBE was distributed into 46 and 65% in the *O*-GlcNAcylated and non-*O*-GlcNAcylated forms, respectively, indicating a preference for S404 phosphorylation in the S400 non-glycosylated form (Figures 4C,D). In contrast, phosphorylation of S202 was increased upon prior *O*-GlcNAcylation. However, S202 phosphorylation level was probably underestimated in p-tau(RBE) due to overlap of S202 and S422 resonances in their non-phosphorylated states while both resonances were resolved in tau-*O*-GlcNAc/p-RBE making phosphorylation changes of S202 and S422 less reliable.

**Figure 5:**
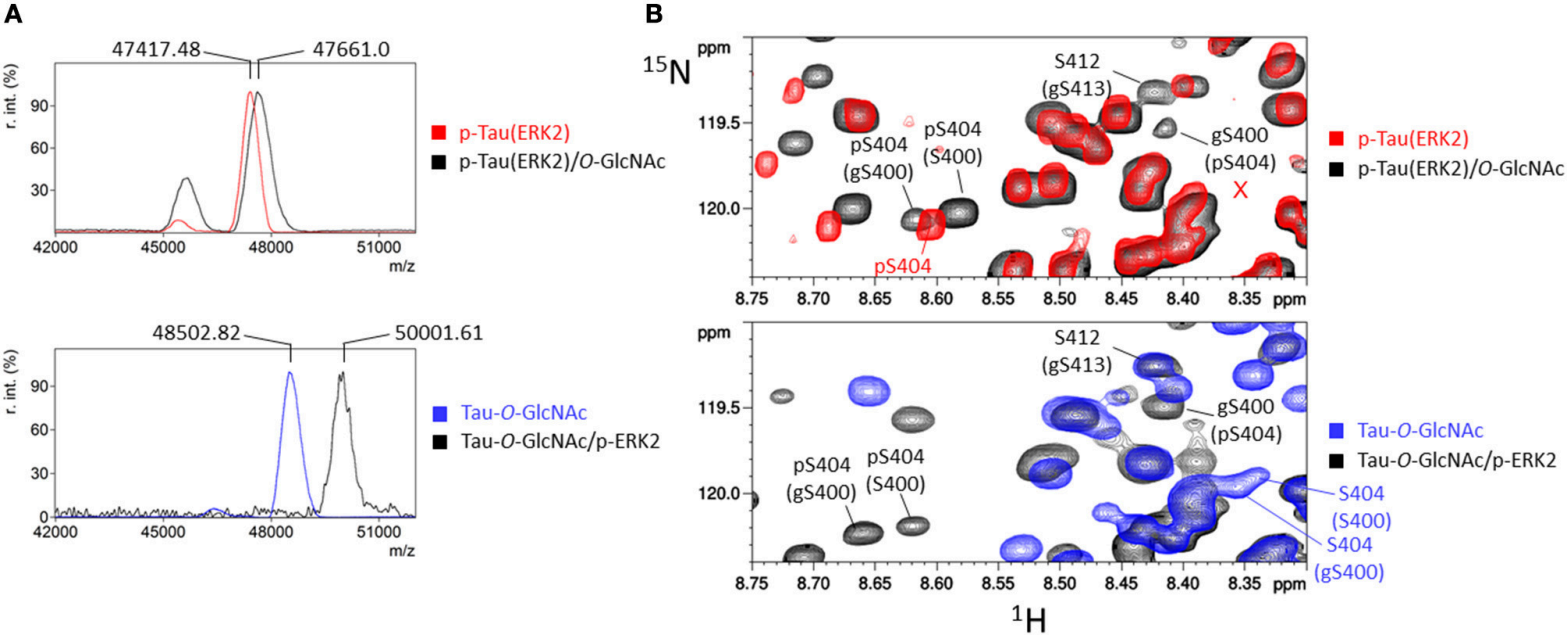
ERK2 phosphorylation of tau and tau-*O*-GlcNAc proteins. **(A)** MALDI-TOF mass spectra of ^15^N-p-tau(ERK2) before (red) and after *O*-GlcNAcylation by OGT (black), and ^15^N/^13^C-tau-*O*-GlcNAc before (blue) and after phosphorylation by ERK2 (black). **(B)** Superimposition of ^1^H-^15^N HSQC spectra of p-tau(ERK2) (red), tau-*O*-GlcNAc (blue) and tau-*O*-GlcNAc/p-ERK2 (black, lower panel) or p-tau(ERK2)/*O*-GlcNAc (black, upper panel) showing the resonances of pS404 and gS400. The red cross marks the absence of S404 resonance indicating a quantitative phosphorylation of S404.

Similarly, the impact of tau *O*-GlcNAcylation on ERK2 phosphorylation which provides hyperphosphorylated tau was studied. No change of phosphorylation level was detected by mass spectrometry upon prior *O*-GlcNAcylation as compared to unmodified tau (Figure 5A and Figure S4). At the site-specific level, almost all phosphorylation sites were quantitatively phosphorylated (Figure S4) whether in tau or tau-*O*-GlcNAc sample. In particular, a quantitative phosphorylation of S404 was reached since no resonance of non-phosphorylated S404 can be detected with S400 being *O*-GlcNAcylated or not (Figure 5B and Figure S6). Furthermore, there is a direct competition for S191 site occupancy which is *O*-GlcNAcylated at 11% in tau-*O*-GlcNAc and phosphorylated at 68% in p-tau(ERK2). Only S400-*O*-GlcNAc resonance was affected by ERK2 phosphorylation indicating that S412 an S413 *O*-GlcNAc sites did not interact with phosphorylation sites. As observed with RBE phosphorylation, a splitting of pS404 resonance upon S400 *O*-GlcNAcylation indicates a local interaction between both PTMs in a site-specific manner (Figures 4D, 5B and Figures S5, S6). In contrast, an absence of splitting of the pS396 resonance suggests that S400-*O*-GlcNAc did not interact with pS396 (Figure S6). Together, these data showed a limited crosstalk between tau *O*-GlcNAcylation and phosphorylation, and suggest that in conditions of hyperphosphorylation, *O*-GlcNAcylation is not able to regulate site-specific phosphorylation level, but only modulates it in conditions of moderate phosphorylation which approximates physiological levels.

## Discussion

**Figure 6:**
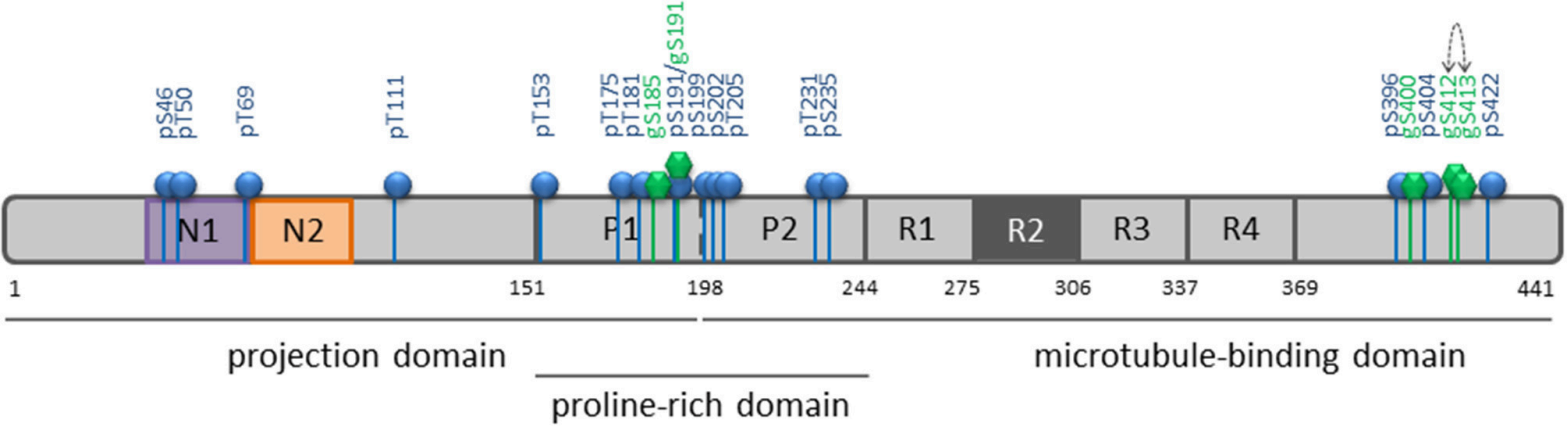
Summary of *O*-GlcNAcylation and phosphorylation sites of tau protein in its longest isoform. ERK2-mediated phosphorylation is depicted by blue bars and balls along the tau primary sequence, and *O*-GlcNAc sites provided by OGT by green bars and hexagons. The dotted arrow indicates mutually exclusive *O*-GlcNAc sites.

Here, we describe for the first time the *O*-GlcNAc pattern of tau protein in its longest isoform using high resolution NMR spectroscopy. Three *O*-GlcNAc sites were found concentrated over a short region of the C-terminus and two *O*-GlcNAc sites in the proline-rich domain, both regions being targeted by multiple phosphorylations (Figure 6). Tau *O*-GlcNAcylation sites (Table S1) found in this study were predicted among others by prediction tools (58, 59). The S400 *O*-GlcNAc site has been previously described in several studies (31, 34, 60–62) and was the sole *O*-GlcNAc site detected in endogenous tau protein from normal and transgenic mice expressing human amyloid precursor protein after enrichment of *O*-GlcNAc proteins while, in contrast, as many as 50 phosphorylation sites were detected (35). This *O*-GlcNAc site was also found in a tau-enriched protein fraction isolated from rat brain in which among 8 sites found in 7 proteins only S400 was identified in tau (62). In addition to S400, Vocadlo and collaborators found in recombinant *O*-GlcNAc modified tau an *O*-GlcNAc site at T123 in the N-terminus which has not been detected here and another one at either S409, S412, or S413 (60). We found both S412 and S413 mono-*O*-GlcNAc species but no di-*O*-GlcNAc form suggesting that both *O*-GlcNAc modifications at vicinal positions are mutually exclusive. Furthermore, S185 and S191 were *O*-GlcNAcylated at lower levels in the proline-rich domain. Taken altogether, these findings refute the statement that tau is an extensively *O*-GlcNAcylated protein (17).

When studying the direct crosstalk between *O*-GlcNAcylation and phosphorylation, we found that hyperphosphorylation obtained with ERK2 and normal phosphorylation (i.e. a phosphorylation level that reproduces physiological phosphorylation of tau) provided by the kinase activity of RBE slightly stimulates tau *O*-GlcNAcylation as detected by a chemoenzymatic labeling strategy. Examination of a phosphorylated/*O*-GlcNAcylated tau sample in which phosphorylation by ERK2 preceded *O*-GlcNAcylation by OGT did not allow finding additional *O*-GlcNAc sites suggesting that phosphorylation increases levels of existing *O*-GlcNAc sites. This finding is in sharp contrast with what has been established in cellular models and in rat brain in which upon perturbations of phosphorylation dynamics, phosphorylation was found to be antagonistic of the *O*-GlcNAc modification (11, 12, 29). Furthermore, hyperphosphorylated forms of tau were found to be significantly less *O*-GlcNAcylated than the non- or less phosphorylated forms (11, 29). Here, we showed that phosphorylation did not inhibit tau *O*-GlcNAcylation by OGT but, in contrast, increasing phosphorylation level improves *O*-GlcNAcylation. Hence, our data argue in favor of an indirect regulation in *in vivo* or *in vitro* models in which tuning the phosphorylation balance could induce a dysregulation of enzymes involved in the *O*-GlcNAc dynamics which in turn, has an impact on the *O*-GlcNAcylation state of tau. Moreover, it has been shown that pharmacological elevation of tau *O*-GlcNAcylation was a potent mechanism to slow down neurodegeneration, reduce tauopathy, and inhibit tau aggregation (31, 34, 36). Our data suggest that hyperphosphorylation of tau is not antagonistic of glycosylation and may rather directly contribute to an increase of tau *O*-GlcNAcylation which could be a salvage mechanism to protect cells from tau toxicity and fibrillar aggregation, two processes which seems to have their origins in tau hyperphosphorylation. This protective mechanism could be impaired in AD brain where the *O*-GlcNAc dynamics is strongly perturbed due to lower glucose metabolism/uptake.

Then, we have investigated the direct effect of *O*-GlcNAcylation on tau phosphorylation provided either by RBE or ERK2 kinase activity. We found that, within the RBE phosphorylation pattern, *O*-GlcNAcylation weakly decreases phosphorylation at a proximal position, S404, but had no significant effect on the overall phosphorylation pattern at the quantitative level. Interestingly, S400 *O*-GlcNAc site is located within the pathological PHF-1 phospho-epitope (pS396/pS404) and interacts with pS404 as both S400 and S404 resonances are affected by the modification of each other, unlike S396 which has no interaction with S400 glycosylation. On the other hand, S400 is the sole *O*-GlcNAc site involved in a crosstalk with phosphorylation. These data suggest that S400 *O*-GlcNAcylation and/or S404 phosphorylation induce a local conformational change allowing a crosstalk between both specific PTMs. In conditions of physiological phosphorylation level, we have noticed that S404 is preferentially phosphorylated in the S400 non-glycosylated form (65% vs. 46% in the S400-*O*-GlcNAc form) but is not inhibited by proximal glycosylation. Interestingly, similar values were found in a short peptide of tau (residues 392–411) phosphorylated by the CDK2/cyclineA3 complex in which S404 phosphorylation level of 61% was reduced to 41% when S400 was *O*-GlcNAcylated (47). Hence, glycosylation of S400 can down-regulate phosphorylation of S404 which is a priming site of GSK3β (44, 47), one of the potential kinases involved in tau hyperphosphorylation associated to tau pathology, and directly compete with S400 phosphorylation preventing from the formation of the PHF-1 phospho-epitope. In contrast, in conditions of hyperphosphorylation, S404 and S396 are almost quantitatively phosphorylated and S400 *O*-GlcNAcylation does not prevent hyperphosphorylation of both sites. Together our data suggest that *O*-GlcNAcylation could be involved in the control of normal phosphorylation but fails to prevent from hyperphosphorylation.

Several studies have shown a reciprocal negative regulation of tau phosphorylation and *O*-GlcNAcylation in cellular models or transgenic mice. In most cases, site-specific tau phosphorylation was increased upon mouse starvation mimicking low glucose metabolism/uptake of AD brain or OGT knock-down, both decreasing protein *O*-GlcNAcylation (11, 18). Conversely, a negative regulation of tau phosphorylation was detected upon increasing *O*-GlcNAcylation with Thiamet-G, a potent OGA inhibitor (36, 61). However, an activation of GSK3β in mouse brain after Thiamet-G injection was observed through an inhibition of AKT phosphorylation (AKT negatively regulates GSK3β via phosphorylation of S9) leading to an increase of site-specific tau phosphorylation (63). Our data giving a quantitative picture of the direct crosstalk between phosphorylation and *O*-GlcNAcylation are contradictory with the findings that *O*-GlcNAcylation of tau has a large effect on site-specific phosphorylation distributed along the entire protein sequence. Hence, this points to an indirect effect on tau phosphorylation resulting from the perturbation of *O*-GlcNAc dynamics *in vitro* or *in vivo* such as an *O*-GlcNAc-mediated regulation of enzymes involved in the phosphorylation balance, e.g. kinases implicated in tau (hyper)phosphorylation (52, 64–67), or other actors in tau pathology such as chaperones and heat-shock proteins (33).

## Author contributions

CS designed research; GB, AK, and FC performed research; GB, AK, BC, and CS contributed new materials, analytic tools; GB and CS analyzed data; IL and CS wrote the paper.

### Conflict of interest statement

The authors declare that the research was conducted in the absence of any commercial or financial relationships that could be construed as a potential conflict of interest.

## Abbreviations

Da: Dalton
gS: *O*-GlcNAc serine
HSQC: heteronuclear single quantum correlation
IPTG: isopropyl-β-D-1-thiogalactopyranoside
MALDI-TOF MS: Matrix-Assisted Laser Desorption-Ionization Time-Of-Flight mass spectrometry
M.W.: molecular weight
*O*-GlcNAc: *O*-β-linked N-acetylglucosamine
pS: phospho-serine
PTM: posttranslational modification
PUGNAc: *O*-(2-Acetamido-2-deoxy-D-glucopyranosylidene)amino-*N*-phenylcarbamate
RBE: rat brain extract
RP-HPLC: reverse phase high performance liquid chromatography
SDS-PAGE: sodium dodecyl sulfate polyacrylamide gel electrophoresis
TAMRA: tetramethylrhodamine
TFA: trifluoroacetic acid
THP: Tris(hydroxypropyl)phosphine.
