# Supplementary material for: Direct Crosstalk Between O-GlcNAcylation and Phosphorylation of Tau Protein Investigated by NMR Spectroscopy
Source: Front Endocrinol (Lausanne). 2018 Oct 16;9:595. doi: 10.3389/fendo.2018.00595 (PMC6198643; doi:10.3389/fendo.2018.00595)
Supplement: Supplementary file 1 [file Data_Sheet_1.docx]

*Supplementary Material*

**Direct crosstalk between *O*-GlcNAcylation and phosphorylation of tau protein investigated by NMR spectroscopy**

**Gwendoline Bourré,^1^ François-Xavier Cantrelle,^1^ Amina Kamah,^1^ Béatrice Chambraud,^2^ Isabelle Landrieu,^1^ Caroline Smet-Nocca^1^***

^1^ Univ. Lille, CNRS UMR8576, Unité de Glycobiologie Structurale et Fonctionnelle, F-59000 Lille

^2^ Univ. Paris XI, UMR 1195 Inserm, Le Kremlin Bicêtre, France

*** Correspondence:** Dr. Caroline Smet-Nocca : [caroline.smet-nocca@univ-lille.fr](mailto:caroline.smet-nocca@univ-lille.fr)

# Supplementary Figures and Tables

## Supplementary Tables

**Table S1**

| ***O*-GlcNAc site** | **sequence** |
| --- | --- |
| **S185** | KTPPS**S**GEPPK |
| **S191** | GEPPK**S**GDRSG |
| **S400** | KSPVV**S**GDTSP |
| **S412** | HLSNV**S**STGSI |
| **S413** | LSNVS**S**TGSID |

**Supplementary Table 1.** Primary sequences around tau *O*-GlcNAc sites identified in this study (indicated in bold red character).

## Supplementary Figures

**Figure S1**


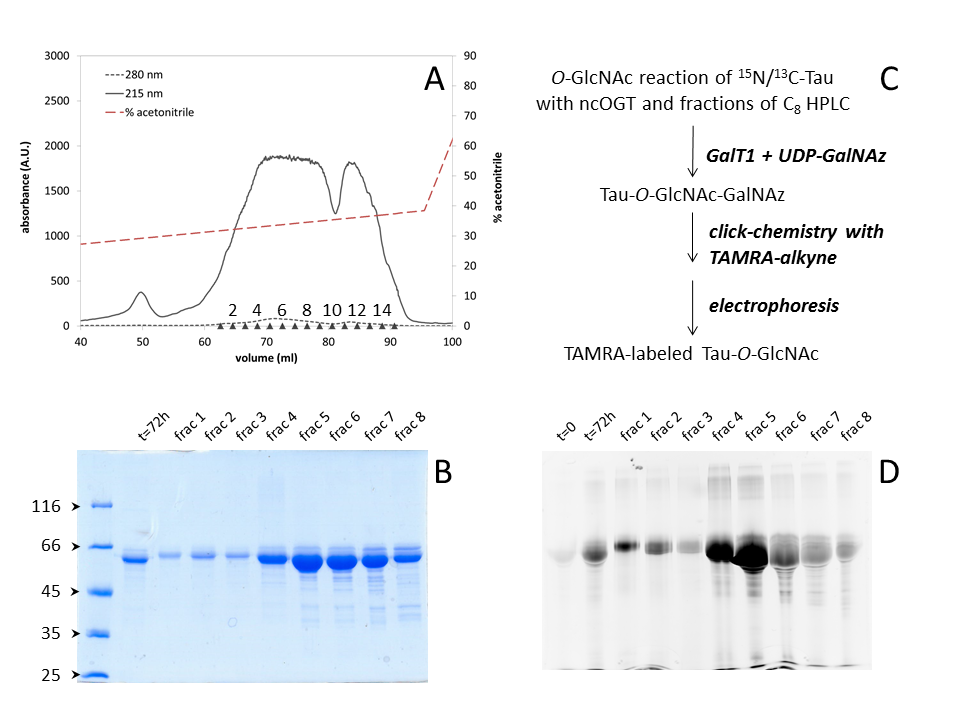


**Supplementary Figure 1.** Purification of tau-*O-*GlcNAc protein. (A) *O-*GlcNAc enrichment by RP-HPLC on C8 column of tau-*O-*GlcNAc after incubation with ncOGT. Absorbance at 280 nm and 215 nm are depicted by grey dotted and solid curves, and linear gradient of acetonitrile by red dotted line. (B) SDS-PAGE analysis of HPLC fractions and the crude *O-*GlcNAc transferase reaction of tau with ncOGT after 72h incubation and heating at 75°C (t=72h) stained with Coomassie dye. (C,D) Protocol of protein *O-*GlcNAc labeling and detection by click-chemistry with TAMRA-alkyne (C) used for the detection of *O-*GlcNAc-enriched tau fractions and the crude *O-*GlcNAc transferase reaction of tau with ncOGT at initial step (t=0) and after 72h-incubation and heating at 75°C (t=72h) (D).

**Figure S2**


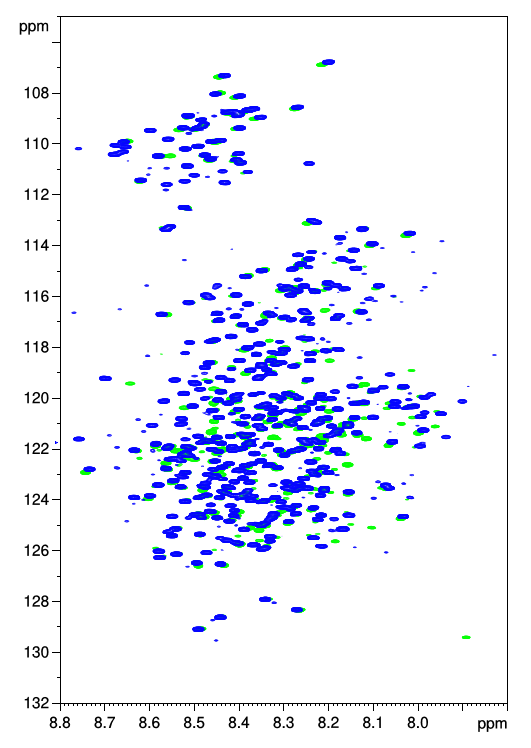


**Supplementary Figure 2.** Superimposition of ^1^H-^15^N HSQC spectra of tau (green) and tau-*O*-GlcNAc (blue).

**Figure S3**


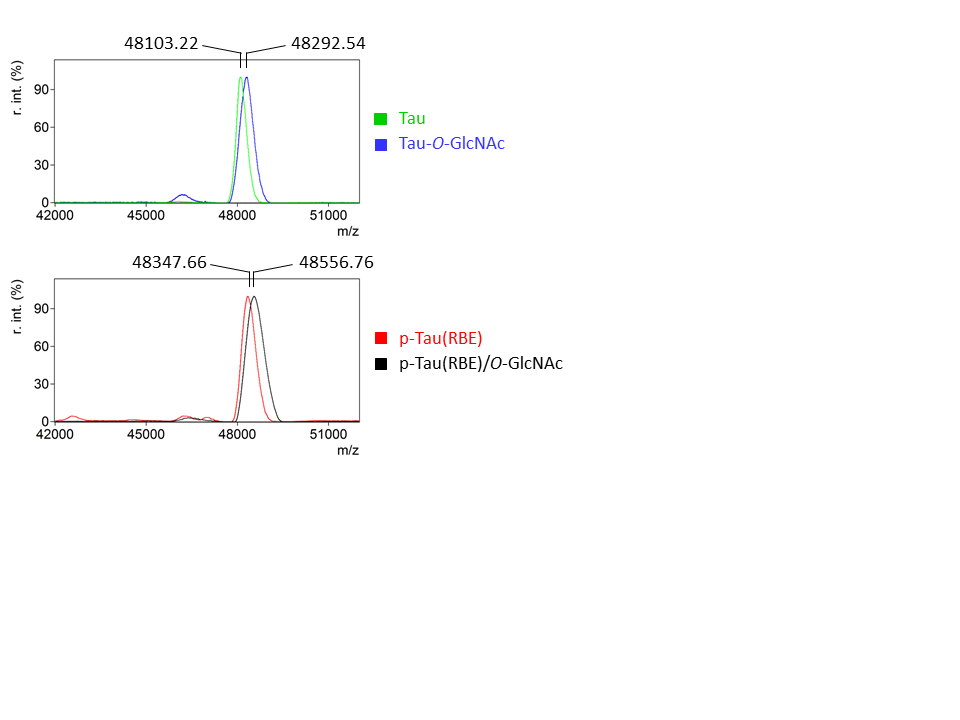


**Supplementary Figure 3.** MALDI-TOF mass spectra of ^15^N-tau before (green) and after *O*-GlcNAcylation by ncOGT (blue), and ^15^N/^13^C-tau phosphorylated by RBE, p-Tau(RBE), before (red) and after *O*-GlcNAcylation by ncOGT (black). *O*-GlcNAcylation reactions were performed at 400μM tau or p-tau(RBE) protein for 48h at 31°C leading to the addition of 0.93 and 1.03 GlcNAc, respectively.

**Figure S4**


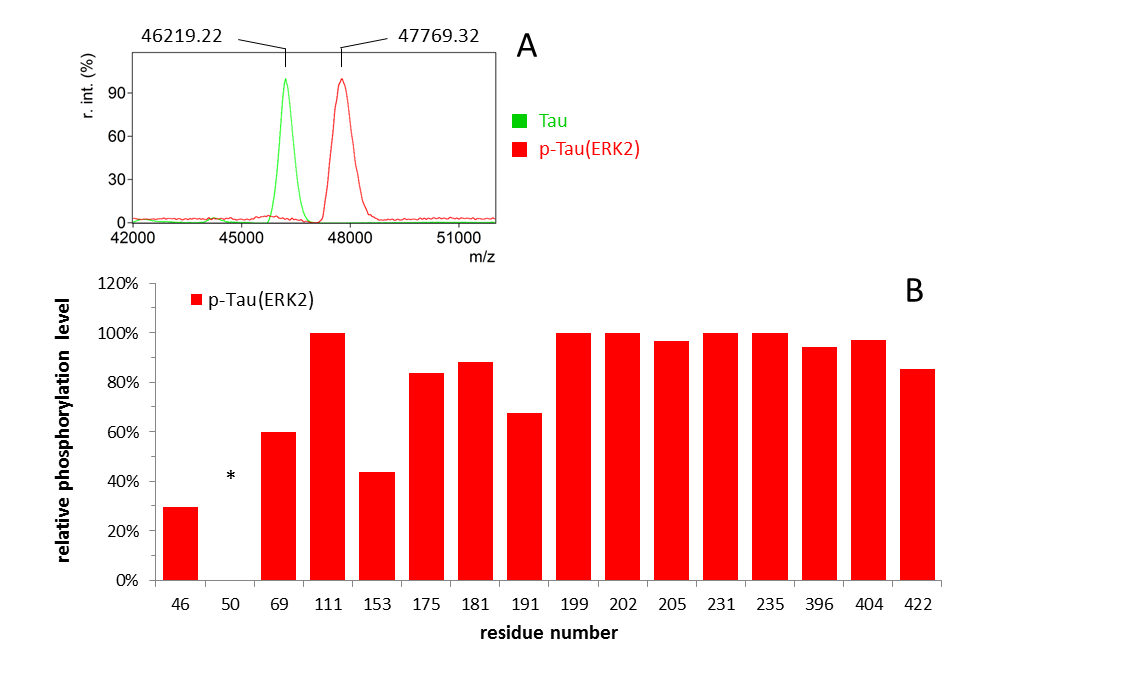


**Supplementary Figure 4.** ERK2 phosphorylation of tau. (A) Overall phosphorylation level of tau measured by mass spectrometry. MALDI-TOF mass spectra of ^15^N-tau (green) and ^15^N-p-tau(ERK2) (red). (B) Relative level of phosphorylation sites in p-Tau(ERK2). Phosphorylation of T50 indicated by an asterisk (*) cannot be determined due to signal overlap of T50 resonance of the non-phosphorylated form.

**Figure S5**


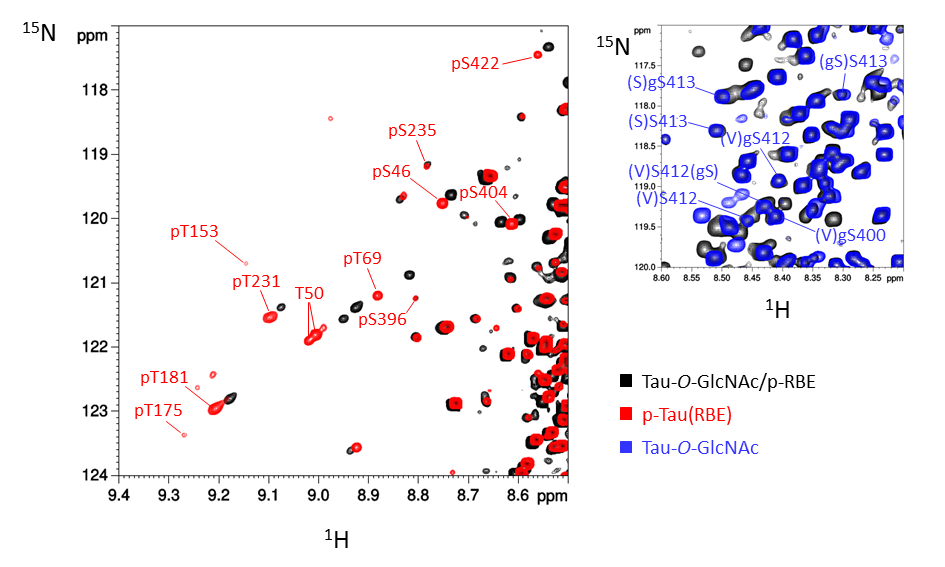


**Supplementary Figure 5.** Superimposition of ^1^H-^15^N HSQC spectra of tau phosphorylated by kinase activity of RBE, p-Tau(RBE) (red), tau-*O*-GlcNAc (blue) and tau-*O-*GlcNAc phosphorylated by RBE, tau-*O-*GlcNAc/p-RBE (black) highlighting the resonances of phospho-residues (left panel) and *O-*GlcNAc residues (right panel).

**Figure S6**


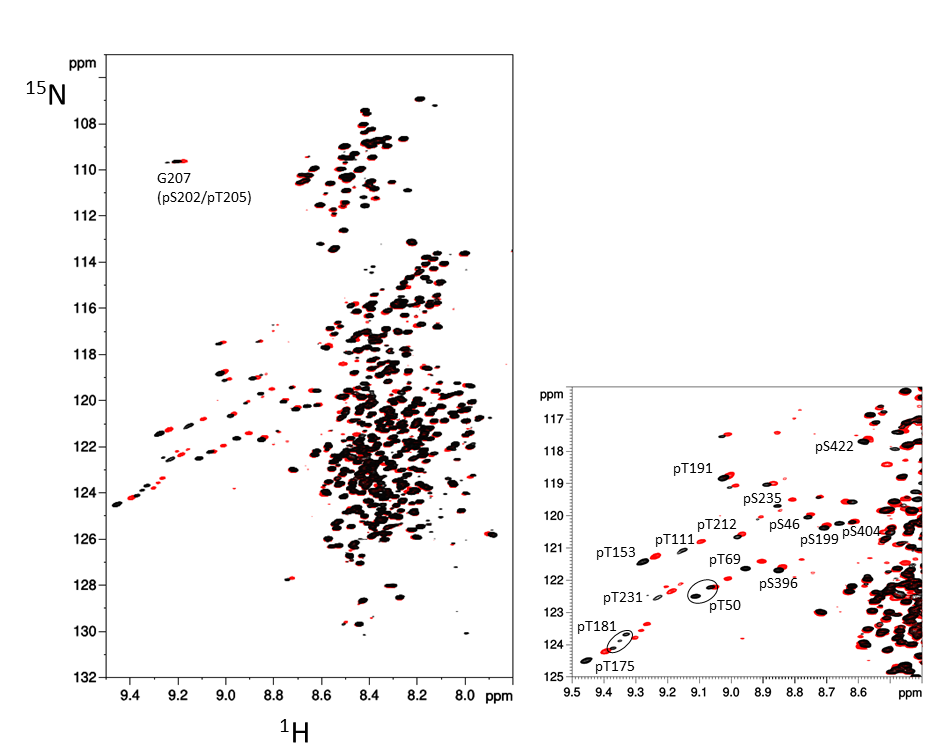


**Supplementary Figure 6.** ERK2 phosphorylation of tau and tau-*O*-GlcNAc. Superimposition of ^1^H-^15^N HSQC spectra of p-Tau(ERK2) (red) and Tau-*O*-GlcNAc/p-ERK2 (black). A zoom on the region of phospho-residue resonances is depicted in the right panel.
